# Felon Manufactory

**Published:** 1857-08

**Authors:** 


### FELON MANUFACTORY.

WHOSE children people our Penitentiaries? They are those of parents who were too indulgent, or too proud, or too indifferent, to bring up their children to some honest trade. Of the six hundred crushed and blasted creatures in the Ohio Penitentiary, whose days are spent in bootless and ignominious toil, and whose narrow night-dungeon is the mute witness of demoniac defiance, or unavailing remorse; of vain curses, fierce and deep, or of feeding on the fires of sharp pointed memories—two-thirds never knew a trade, five-sixths are unable to read or write. How much of truth is there in Franklin's reputed saying, "He who fails to teach his child a trade, teaches him to become a scoundrel!"

